# Downregulation of GPX8 in hepatocellular carcinoma: impact on tumor stemness and migration

**DOI:** 10.1007/s13402-024-00934-w

**Published:** 2024-04-12

**Authors:** Chen-Yang Tao, Xiao-Ling Wu, Shu-Shu Song, Zheng Tang, Yu-Fu Zhou, Meng-Xin Tian, Xi-Fei Jiang, Yuan Fang, Gui-Qi Zhu, Run Huang, Wei-Feng Qu, Jun Gao, Tian-Hao Chu, Rui Yang, Jia-Feng Chen, Qian-Fu Zhao, Zhen-Bin Ding, Zhi Dai, Jian Zhou, Wei-Ren Liu, Ying-Hong Shi, Jia Fan

**Affiliations:** 1 Department of Liver Surgery and Transplantation, Liver Cancer Institute, Zhongshan Hospital, Key Laboratory of Carcinogenesis and Cancer Invasion of Ministry of Education, Fudan University, Shanghai, China; 2 Research Unit of Liver cancer Recurrence and Metastasis, Chinese Academy of Medical Sciences, Beijing, China; 3 Department of Biochemistry and Molecular, School of Basic Medical Sciences, Fudan University, Shanghai, China; 4 Department of Immunology and Pathogenic Biology, School of Basic Medical Sciences, Shanghai University of Traditional Chinese Medicine, Shanghai, China

**Keywords:** GPX8, Hepatocellular carcinoma, Tumor stemness, Migration, Hsc70

## Abstract

**Purpose:**

GPX8, which is found in the endoplasmic reticulum lumen, is a member of the Glutathione Peroxidases (GPXs) family. Its role in hepatocellular carcinoma (HCC) is unknown.

**Methods:**

Immunohistochemical staining was used to detect the protein levels of GPX8 in HCC tissue microarrays. A short hairpin RNA lentivirus was used to knock down GPX8, and the main signaling pathways were investigated using transcriptome sequencing and a phosphorylated kinase array. The sphere formation assays, cloning-formation assays and cell migration assays were used to evaluate the stemness and migration ability of HCC cells. Identifying the GPX8-interacting proteins was accomplished through immunoprecipitation and protein mass spectrometry.

**Results:**

The GPX8 protein levels were downregulated in HCC patients. Low expression of GPX8 protein was related to early recurrence and poor prognosis in HCC patients. GPX8 knockdown could enhance the stemness and migration ability of HCC cells. Consistently, Based on transcriptome analysis, multiple signaling pathways that include the PI3K-AKT and signaling pathways that regulate the pluripotency of stem cells, were activated after GPX8 knockdown. The downregulation of GPX8 could increase the expression of the tumor stemness markers KLF4, OCT4, and CD133. The in vivo downregulation of GPX8 could also promote the subcutaneous tumor-forming and migration ability of HCC cells. MK-2206, which is a small-molecule inhibitor of AKT, could reverse the tumor-promoting effects both in vivo and in vitro. We discovered that GPX8 and the 71-kDa heat shock cognate protein (Hsc70) have a direct interaction. The phosphorylation of AKT encouraged the translocation of Hsc70 into the nucleus and the expression of the PI3K p110 subunit, thereby increasing the downregulation of GPX8.

**Conclusion:**

The findings from this study demonstrate the anticancer activity of GPX8 in HCC by inactivating the Hsc70/AKT pathway. The results suggest a possible therapeutic target for HCC.

**Supplementary Information:**

The online version contains supplementary material available at 10.1007/s13402-024-00934-w.

## Introduction

Hepatocellular carcinoma (HCC) is a primary liver cancer type that remains a significant global health concern due to the high mortality rates and limited effective therapies [1, 2]. HCC commonly metastasizes (spreads to other organs) and complicates treatments often lead to poor outcomes for patients [3, 4]. Cancer stem cells (CSCs) are a subset of cells that initiate tumors, resist conventional treatments and contribute to metastasis [5]. Thus, understanding the mechanisms of HCC progression, specifically those involving CSCs, is of paramount importance.

Glutathione Peroxidase 8 (GPX8) is one member of the Glutathione Peroxidase (GPX) family, which play a vital role in maintaining redox balance, thereby protecting cells from oxidative damage [6, 7]. It has been reported that GPX8 plays a crucial role in maintaining an aggressive phenotype in breast cancer cells through the IL-6/STAT3 axis [8]. Additionally, a correlation between GPX8 and poor prognosis have been reported in various cancer types [8–11]. However, the function and action mechanism of GPX8 in HCC remain unclear.

This study aims to clarify the relationship between GPX8 and clinical outcomes in HCC, mainly focusing on early recurrence and patient prognosis. The findings from this study suggest that GPX8 is downregulated in HCC patients, a trend that is associated with an increased risk of early tumor recurrence and lower chances of patient survival. GPX8 knockdown could significantly enhance the stemness and migration abilities of HCC cells via activating PI3K/AKT signaling. When GPX8 expression reduces, the expression of tumor stemness markers, such as KLF4, OCT4, and CD133 tend to increase. This indicates enhanced tumorigenic and migratory capabilities of HCC cells. A noteworthy finding from the current research is the interaction between GPX8 and the 71-kDa heat shock cognate protein (Hsc70). Downregulation of GPX8 appears to accelerate the nuclear translocation of Hsc70 and subsequent upregulation of the PI3K p110 subunit. This ultimately causes increased AKT phosphorylation, which is a key regulator of cell stemness and migration.

The current research underscores the critical need for exploring the role of the endoplasmic reticulum-associated protein GPX8 in cancer biology. We anticipate that the insights from this study will facilitate the advancement of superior treatment approaches for HCC, thereby enhancing prognosis and quality of life for patients.

## Materials and methods

### Human sample and TMA assay

Tumor specimens for tissue microarrays were obtained from 354 HCC patients who underwent surgical procedures between April 2005 and September 2008 at the Department of Liver Surgery, Zhongshan Hospital of Fudan University. All patients were duly informed about the purpose of the study and consented to the use of their samples. Tissue microarray (TMA) was conducted as previously described [12, 13]. Evaluation of immunohistochemical staining was also carried out as previously described [14]. All images were analyzed using a computer-automated method (Image-pro plus 6.0, Media Cybernetics, Silver Springs, MD, USA) [15].

### Cell culture and animals model

HCC cell lines L-02, MHCC97-L, MHCC97-H, HCCLM3, SNU-449, and Huh7 were from Song Shushu (Zhongshan Hospital, Fudan University). L-02, MHCC97-L, MHCC97-H, HCCLM3, and Huh7 was cultured in DMEM medium supplemented with 10% FBS, 100 unit/ml streptomycin, 100 μg/ml penicillin. SNU-449 was cultured in RMPI-1640 medium supplemented with 10% FBS, 100 unit/ml streptomycin, 100 μg/ml penicillin. and all cell lines were incubated at 37 °C in a humidified atmosphere with 5% CO_2_. All cell culture reagents were obtained from Gibco (Invitrogen, USA).

male BALB/c nude mice weighed 18–20 g and aged 4–6 weeks were purchased from the Shanghai Model Organisms Center, Inc. (Shanghai, China). All model mice were maintained in specific pathogen-free conditions. Humane care of animals was objected to the “Guide for the Care and Use of Laboratory Animals” criteria of the National Academy of Science (National Institute of Health publication 86–23, revised 1985).

Limiting dilution Xenograft arrays evaluate cancer cells’ tumorigenic capacity, making them useful for studying cancer stem cells. Huh7 cells are serially diluted (from 10^7^ to 10^5^ cells) and implanted into nude mice to evaluate tumor formation.

For the mouse model of liver metastasis, each mouse was primed with 200 µL of SNU-449 cell suspension by tail intravenous injection. Two weeks later, intraperitoneal injections of MK-2206 (40 μg/g of body weight) or DMSO were given to all the mice twice a week for a total of two weeks. After that, they were killed, and the liver tissue was removed and preserved in a paraformaldehyde solution to create tissue slices.

### Quantitative real-time PCR (qRT-PCR)

Total cellular RNA extraction was performed using a RNeasy mini kit (Qiagen, Germany) and cDNA was synthesized using the Quantitect Reverse Transcription Kit (Qiagen, Germany) according to the manufacturer’s instructions. Target genes were quantified using FastStart Universal SYBR Green Master (Roche diagnostics, Germany) and DNA amplification was carried out using a LightCycler 480 (Roche Diagnostics, Germany). The relative quantities of target gene mRNAs compared to an internal control were determined using the ΔCq method. PCR conditions were as follows: 5 min at 95 °C, followed by 40 cycles of 95 °C for 10 s and 60 °C for 60 s. GAPDH was used as an internal control. Primers and probes are listed in Table S1.

### Western blot analysis

Protein extraction from the tissue or cell samples was performed using radioimmunoprecipitation assay (RIPA) buffer supplemented with a protease and phosphatase inhibitor cocktail. The protein concentrations were determined using a bicinchoninic acid (BCA) protein assay kit.

Equal amounts of proteins were separated by sodium dodecyl sulfate-polyacrylamide gel electrophoresis (SDS-PAGE) and then transferred onto polyvinylidene fluoride (PVDF) membranes. The membranes were blocked with 5% non-fat milk in Tris-buffered saline with 0.1% Tween 20 (TBST) for 1 hour at room temperature to prevent non-specific binding.

The membranes were then incubated overnight at 4 °C with primary antibodies specific to the target proteins. After washing with TBST, the blots were incubated with horseradish peroxidase (HRP)-conjugated secondary antibodies for 1 hour at room temperature. The blots were washed again with TBST and the protein bands were visualized using an enhanced chemiluminescence (ECL) detection system.

The intensity of the protein bands was quantified using image analysis software and normalized to the intensity of the loading control, typically beta-actin or glyceraldehyde 3-phosphate dehydrogenase (GAPDH).

The antibodies used in this study are listed in Supplementary Table S5.

### Colony formation assay

Once they reached a diameter of 100 μm, HCC spheres were collected through gentle centrifugation, dissociated with trypsin-EDTA (Invitrogen, USA), and mechanically disrupted with a pipette. The resulting cells were gently centrifuged to remove trypsin. Single cells were seeded in DMEM with 10% FBS (Gibco, USA) at a density of 2000 cells per well in a 6-well plate (Corning, USA). Parental Huh7 cells were seeded at the same density as a control population to evaluate colony-forming capacity. After two weeks, the colony-forming ability was assessed by counting the number of colonies (> 70 cells) under a microscope after staining with crystal violet (Sigma-Aldrich, USA). Representative images were photographed using an Olympus LX-71 fluorescence microscope. Experiments were performed in triplicate.

### Sphere-forming assay

Serum-free medium for sphere culture was composed of DMEM/F12 medium supplemented with 100 IU/ml penicillin, 100 μg/ml streptomycin, 20 ng/ml human recombinant epidermal growth factor, 20 ng/ml human recombinant basic fibroblast growth factor, 1% nonessential amino acids, 1% GlutaMax, 2% B27 supplement (Invitrogen, USA), and 1% methylcellulose (Sigma, USA). HCC cells were cultured at a density of 1000 cells/ml; when spheres reached a diameter of 100 μm, the sphere-forming efficiency was calculated and spheres were collected for further use.

### Transwell assay

Transwell assays were used to assess migration activity. The cells were collected and washed with 1x PBS. To conduct migration tests, 5 × 10^4^ cells were seeded in an upper chamber with a non-coated membrane (24-well insert, pore size 8 μm; Corning, USA) in DMEM containing 1% FBS. The lower chambers contained DMEM with 10% FBS as a chemoattractant. Cells were incubated at 37 °C for 24 h. Cells that had migrated or penetrated the bottom surface of the membrane were preserved with 4% methanol and then stained with crystal violet. Stained cells were counted in ten randomly selected 100X microscopic areas. All experiments were conducted in triplicate. The unpaired two-tail student’s t-test was used for comparison.

### Phosphorylation kinase array

The Proteome Profiler Human Phospho-Kinase Array Kit (R&D Systems) was used to simultaneously assess the phosphorylation status of various kinases. Cell lysates were prepared post-stimulation and their protein concentrations determined using a BCA protein assay kit. The array membranes were blocked with array buffer 1 for 1 h, then incubated with 300 μg of cell lysates overnight at 4 °C. After washing, the membranes were further incubated with a cocktail of biotinylated detection antibodies, followed by a horseradish peroxidase-conjugated streptavidin incubation. Protein spots were visualized using a chemiluminescent detection reagent, and signal intensities were quantified using a digital imaging system and image analysis software. The relative phosphorylation levels of the kinases were determined by comparing the signal intensities of individual kinases with the control. Experiments were performed in triplicate, with data expressed as mean ± standard deviation (SD). Statistical significance was determined using a two-tailed t-test, with a p-value of less than 0.05 considered statistically significant.

### Immunoprecipitation (IP)

Cells were transfected as mentioned and collected using immunoprecipitation lysis buffer (Beyotime, Shanghai, China). Equal volumes of cell lysis were then treated for 6 h at 4 °C with moderate rotation with FLAG antibody (1:200; Sigma) immobilized onto Protein G-Sepharose beads. The beads were afterwards washed with lysis buffer three times. Following addition of SDS-PAGE sample loading buffer and subsequent boiling, the beads were centrifuged to obtain supernatant for western blot.

### Nucleoplasmic separation arrays

The Nuclear and Cytoplasmic Protein Extraction Kit (Beyotime) was used to collect and lyse cells in accordance with the manufacturer’s instructions. Using a different lysis buffer and centrifugation, the nuclear and cytoplasmic components of the lysate were separated. Western blot was utilized to analyze the proteins present in the arrays.

### Statistical analysis

Differences between the two groups were analyzed using a two-tailed t-test, while a two-way analysis of variance (ANOVA) was employed to evaluate the statistical significance of two-factor interactions across multiple time points. The X-tile software was used to determine the optimal cut-off point for continuous variables. The cut-off point was subsequently used to divide HCC patients into groups of high and low GPX8 expression. The survival analysis was conducted using the Kaplan-Meier method with a log-rank test to assess risk factors for overall survival (OS), disease free survival (DFS) and recurrence free survival (RFS) in HCC patients. Furthermore, a multivariate Cox regression analysis was performed to identify predictors of prognosis. All data were analyzed using the SPSS software (version 24.0, SPSS Inc.). Statistical significance was considered when the *P*-value was below 0.05 (*P* < 0.05).

## Results

### Reduced GPX8 protein expression predicts a poor prognosis in HCC patients

**Fig. 1:**
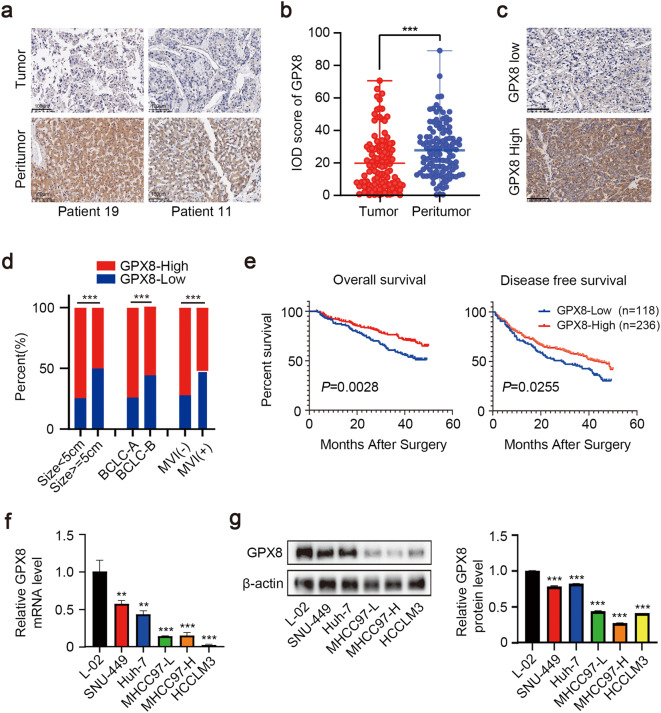
Reduced GPX8 protein expression indicates a poor prognosis in patients with HCC. **a** Representative images of IHC staining of HCC tumor microarrays (TMA) with different GPX8 expression levels. Scale bars, 100 μm. T, tumor. P, peritumor. **b** Analysis of the relative expression of GPX8 in tumor and peritumor tissues. IOD, integral optical density. **c** Representative images of IHC staining of HCC samples with different GPX8 levels. Scale bars, 100 μm. **d** Analysis of the different expression of GPX8 in clinical features. BCLC, Barcelona Clinic Liver Cancer Staging System. MVI, Microvascular invasion. **e** Kaplan-Meier curves were constructed to investigate the relationship between GPX8 expression and both overall survival (OS) (left panel) and disease-free survival (DFS) (right panel) of patients from HCC-TMA datasets. The trisection value is used as the cut-off threshold to classify GPX8 expression as “high” or “low.” **f** qRT-PCR analysis of GPX8 expression in human L02 hepatocytes and five established HCC cell lines (SNU449, Huh7, 97 L, 97 H, and LM3). **g** Immunoblots (upper panel) and relative quantitative analysis (lower panel) of GPX8 levels in L02 and established HCC cell lines. For all the experiments described above, the data in B, D, F, and G are presented as the means ± standard deviations (SDs), and three independent experiments (N = 3) were performed in triplicates. *P < 0.05; **P < 0.01; and ***P < 0.001

To explore the role of GPX8 in HCC, we firstly examined GPX8 expression in HCC microarrays which included 118 clinical samples by immunohistochemistry (IHC) staining. And results revealed that the protein expression of GPX8 was significantly lower in HCC tissues than that in paired peritumor tissues (Fig. 1b). The trisection value served as the cut-off threshold for categorizing samples as GPX8 high expression group or GPX8 low expression group, and the representative images were shown in Fig. 1. Further analysis indicated that patients with advanced clinical characteristics, such as tumor size larger than 5 cm, Barcelona Clinic Liver Cancer Stage B, and positive microvascular invasion, had significantly higher percentages of GPX8 low expression (Fig. 1). IHC staining was then performed on more HCC TMA to increase the sample size to 354. And according to Kaplan-Meier analysis for these samples, patients with high levels of GPX8 had a better prognosis (Fig. 1). Chi-square test demonstrated that the positive rate of alpha fetoprotein (AFP) in the GPX8 low expression group was dramatically higher than that in GPX8 high expression group (Supplementary Table S1). A multivariate Cox proportional hazards model revealed that patients who owned high GPX8 expression exhibited a significantly lower hazard ratio compared to patients whose GPX8 expression was low (Supplementary Table S2). And GPX8 protein levels served as an independent predictor of overall survival for HCC patients (Supplementary Table S2).

Furthermore, GPX8 was detected in 5 kinds of HCC cell lines, together with a normal human hepatocyte cell line. The findings from quantitative real-time polymerase chain reaction (qRT-PCR) and immunoblot analyses indicated that both the mRNA and protein levels of GPX8 in these HCC cell lines were obviously lower compared with the normal L02 cell line (Fig. 1f, g). Taken together, GPX8 is significantly downregulated in HCC and low GPX8 protein expression may be a risk factor for HCC.

### GPX8 knockdown promotes the malignant phenotype of HCC cells in vitro

**Fig. 2:**
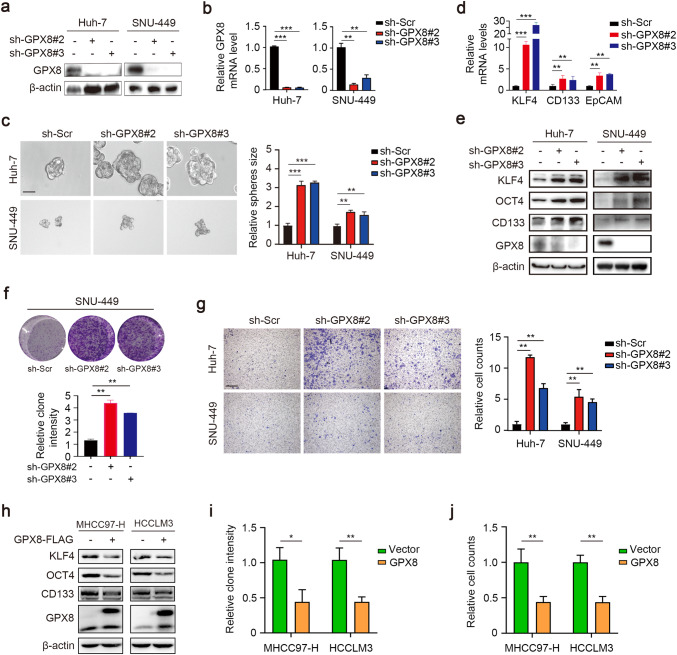
GPX8 knockdown promotes the malignant phenotype of HCC cells in vitro. **a**, **b**, Immunoblotting (**a**) and qRT-PCR (**b**) analyses were performed in SNU449 and Huh7 cells that had been stably infected with lentivirus containing the scramble shRNA (sh-Scr) or GPX8 knockdown shRNA (sh-GPX8). **c** A tumor-sphere forming assay was performed in SNU449 or Huh7 cells for four and seven days, respectively. The representative images (left panel) and the relative numbers of spheres (right panel) were shown. **d** qRT-PCR analysis of KLF4, CD133, and EpCAM mRNA levels in SNU449 cells infected with sh-Scr or sh-GPX8. **e** Immunoblots show the levels of KLF4, OCT4, CD133, and GPX8 in SNU449 or Huh7 cells infected with sh-Scr or sh-GPX8. **f** A clone formation assay was performed on the SNU449 cells that were infected with sh-Scr or sh-GPX8 for a week; the representative images (upper panel) and the quantitative analysis of the clone number (lower panel). **g** A transwell assay was performed in SNU449 or Huh7 cells. The representative images (left panel) and the relative numbers of migrating cells (right panel) were shown. **h** Immunoblots show the levels of KLF4, OCT4, CD133, and GPX8 in the MHCC97-H and HCCLM3 cell lines transfected with the Flag-tagged GPX8 plasmid. **i** A clone formation assay was performed in the MHCC97-H and HCCLM3 cell lines transfected with the flag-tagged GPX8 plasmid, and the quantitative analysis of the clone numbers was shown. **j** A transwell assay was performed in the MHCC97-H and HCCLM3 cell lines transfected with the flag-tagged GPX8 plasmid, and the relative numbers of migrating cells were shown. The data for the above experiments were presented as means ± standard deviations (SDs), and three independent experiments (N = 3) were performed in triplicates. *P < 0.05; **P < 0.01; and ***P < 0.001

To better understand the biological role of GPX8 in HCC, GPX8 stable knockdown Huh7 and SNU-449 cell lines were established which owned relatively high expression of GPX8 (Fig. 1f, g). MHCC97-H and HCCLM3 cell lines with relatively low expression of GPX8 were utilized to perform GPX8 overexpression experiments (Fig. 1f, g). GPX8 was successfully knocked down in both Huh7 and SNU-449 cell lines, as validated by Western blot and qRT-PCR (Fig. 2a, b). Surprisingly, we found that both Huh7 and SNU-449 cell lines significantly enhance the sphere-forming ability of HCC cells (Fig. 2c). This phenomenon suggests that GPX8 may enhance the stemness of HCC cells, thus we investigated the expression of several typical cancer stemness markers. qRT-PCR results showed that GPX8 knockdown significantly elevated mRNA levels of CD133, KLF4, and EpCAM (Fig. 2d). Western blot analysis revealed that GPX8 knockdown significantly raised the protein levels of KLF4, OCT4, and CD133 (Fig. 2e). To find out if GPX8 knockdown enhances HCC cell malignancy, several cellular functional studies were performed. We found that GPX8 knockdown also increased the ability of HCC cell lines to migrate and form clones (Fig. 2f, g).

To restore low GPX8 expression, the MHCC97-H and HCCLM3 cell lines were transfected with the Flag-tagged GPX8 plasmid. Our findings that overexpress GPX8 reduced the protein levels of KLF4, OCT4, and CD133 were in consistent with the results of knockdown experiments (Fig. 2h). Meanwhile, in the MHCC97-H and HCCLM3 cell lines, which have a more aggressive malignant phenotype in wild-type, the abilities of clone formation and migration were impaired by GPX8 overexpression (Fig. 2 i-j). Overall, these results support the hypothesis that GPX8 deficiency contributes to the malignant phenotype of HCC in vitro.

### In vitro or in vivo, GPX8 knockdown promotes the tumor malignant phenotype through the PI3K-AKT signaling pathway in an AKT-dependent manner

**Fig. 3:**
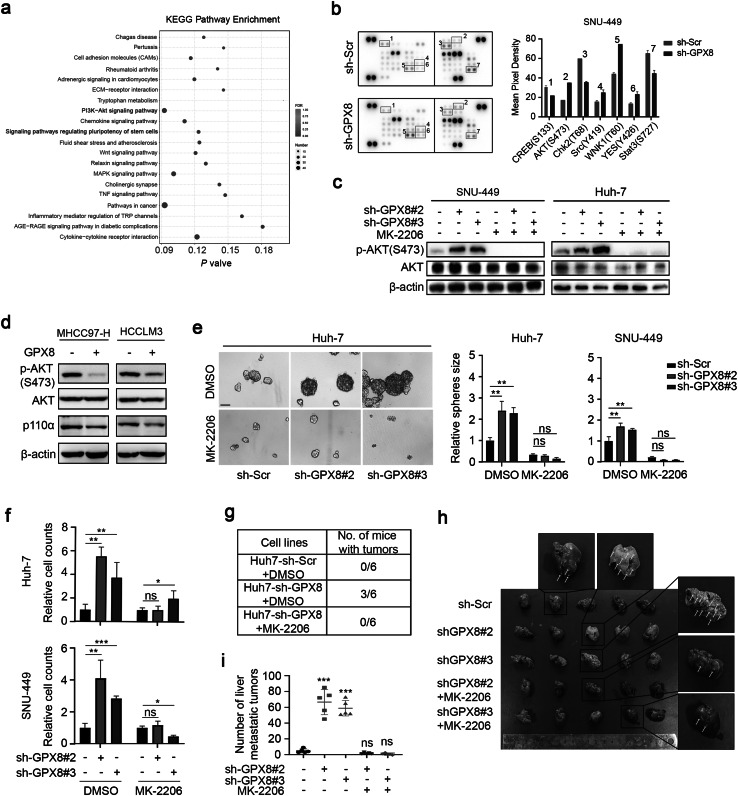
GPX8 knockdown promotes the PI3K-AKT signaling pathway in an AKT-dependent manner in vitro or in vivo. **a** A KEGG pathway enrichment analysis of SNU-449 stable cell lines transcriptome sequencing (sh-GPX8 vs sh-Scr). Bold font, the major up-regulated signaling pathway. **b**, Phosphorylated kinase microarrays were performed in SNU449 cells to investigate the downstream signal pathway of GPX8 knockdown. The representative images of microarrays (left) were displayed, and the red boxes indicate that the phosphorylation levels are significantly changing. The quantitative analysis (right) of these sites was shown. **c** immunoblots show AKT and AKT phosphorylation (p-AKT) levels in SNU449 or Huh7 cells that were treated with DMSO or MK-2206 for two hours. **d** immunoblots show AKT, p110α and AKT phosphorylation (p-AKT) levels in the MHCC97-H and HCCLM3 cell lines transfected with the flag-tagged GPX8 plasmid. **e**, **f** A tumor-sphere-forming assay was performed in SNU449 or Huh7 cells that were treated for four or seven days with DMSO or MK-2206. The representative images of Huh7 cell (left panel) and the relative numbers of spheres (right panel) were shown separately. **f** A transwell assay was performed in SNU449or Huh7 cells. The relative numbers of migrating cells were shown separately. **g** A limited number (2 × 10^5^) of Huh7 cells that were infected with sh-Scr or sh-GPX8 were subcutaneously injected into the right flanks of male BALB/c nude mice (n = 6 mice per group), after which they were treated with DMSO or MK-2206 that was performed via intraperitoneal injection every two days up to two weeks. **h, i**, SNU449 cells infected with sh-Scr or sh-GPX8 were intravenously injected into the tail vein of male BALB/c nude mice (n = 5 mice per group), after which they were treated with DMSO or MK-2206, which was performed via intraperitoneal injection every two days up to two weeks. The quantitative analysis (**i**) of the number of tumor metastatic clusters in the liver and the representative images (**h**) were shown. The above experiments’ data are presented as means ± standard deviations (SDs), and three independent experiments (N = 3) were performed in triplicates. *P < 0.05; **P < 0.01; and ***P < 0.001

The signaling pathways that may be primarily affected by GPX8 knockdown were investigated using Transcriptome sequencing and phosphorylated kinase microarrays (Fig. 3a, b). After GPX8 knockdown, the transcriptome sequencing data showed that the PI3K-AKT signaling pathways were significantly upregulated (Fig. 3a). In the meantime, the phosphorylated kinase microarrays demonstrated the significant amplification of AKT^Ser473^ phosphorylation (Fig. 3b). Together, these results suggest that PI3K-AKT may be the key downstream signaling pathway that is regulated by GPX8.

Further investigations were made to verify if GPX8 affected the kinase activity of AKT in HCC cells. As expected, immunoblotting showed that GPX8 knockdown enhanced the phosphorylation of AKT^Ser473^ in Huh7 and SNU449 cells (Fig. 3c), and this effect was reversed by the AKT inhibitor MK-2206 (Fig. 3c). Whereas overexpression of GPX8 reduced the AKT^Ser473^ phosphorylation and p110α expression in the MHCC97-H and HCCLM3 cell lines (Fig. 3d). Likewise, MK-2206 can reverse the effect of GPX8 knockdown, thereby encouraging the malignant phenotype of HCC cells, including the capacity for sphere formation (Fig. 3e) and migration (Fig. 3f).

In vivo experiments were used to confirm the tumor-promoting effect of GPX8 knockdown, in addition to determining whether it is dependent on AKT phosphorylation or not. According to limiting dilution xenograft assays, GPX8 knockdown significantly enhanced the formation of tumors in Huh7 cells, compared to control cells, and this effect can be reversed by MK-2206 (Fig. 3g). With SNU449 cells, this study discovered that it was difficult to form tumors in xenograft models via subcutaneous injection under various experimental conditions. However, the liver metastasis model in nude mice can be established through injecting SNU-449 cells through the tail vein, allowing us to assess the metastatic and tumorigenic capacities of HCC cell lines in this experiment. Similar to the results of Huh7 cells, the GPX8 knockdown group discovered significantly more micro metastasis in the mice’s livers than the control group, four weeks after injecting SNU449 cells into mice’s tail veins. This effect was also significantly reversed by MK-2206 (Fig. 3h, i). Put together, these results showed that in vitro or in vivo, GPX8 knockdown promotes the tumor malignancy phenotype through the PI3K-AKT signaling pathway in an AKT-dependent manner.

### GPX8 knockdown increased Hsc70 nuclear translocation

**Fig. 4:**
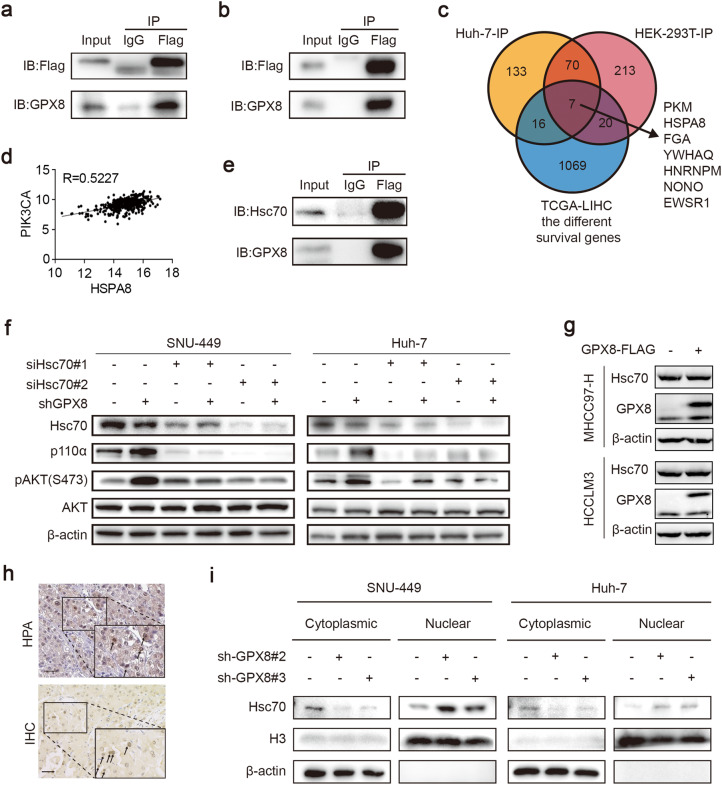
Hsc70 nuclear translocation is reduced by GPX8 binding. **a**, **b** SNU449 lysates (**a**) or HEK-293T lysates (**b**) were immunoprecipitated with anti-IgG and anti-FLAG antibodies and immunoblotted with the indicated antibodies. **c** The schematic diagram of the screening process for the key GPX8 binding protein. **d** Correlation analysis of Hsc70 with the expression of the PI3K p110α subunit in the TCGA-LIHC database. **e** Immunoblotting was performed to validate the screening results of LC-MS. **f** Immunoblots show the levels of AKT, pAKT, PI3K p110α, and Hsc70 in SNU449 or Huh7 cells that were transfected with the control siRNA (si-CTL) or Hsc70 siRNAs (si-Hsc70). **g** Immunoblots show the levels of Hsc70 and GPX8 in the MHCC97-H and HCCLM3 cell lines transfected with the flag-tagged GPX8 plasmid. **h** the representative images of IHC staining for Hsc70 from the Human Protein Atlas (upper) and from HCC samples of our cohort (lower). The arrows indicate the nuclei of cells with high Hsc70 expression. Scale bars, 100 μm. **i** immunoblots show the level of Hsc70 in the cytoplasm and cell nucleus after nucleoplasmic separation arrays

To generate a GPX8 binding protein library and investigate the potential mechanism by which GPX8 inhibited the progression of HCC, liquid chromatography with tandem mass spectrometry (LC-MS/MS) was used to analyze the pull-down proteins after IP. Antibodies against Flag were employed as bait in SNU449 or HEK-293T cell lines transfected with Flag-tagged GPX8 overexpression plasmid (Supplementary Table S2). Meanwhile, immunoblotting was used to validate the IP experiments (Fig. 4a, b). A total of 77 potential proteins that could bind to the GPX8 were identified (Fig. 4c). To further narrow down the list of potential proteins, a checklist of the genes that are most associated with patient survival in the TCGA-LIHC dataset was obtained from GEPIA (http://gepia.cancer-pku.cn). Finally, seven candidate molecules were identified (PKM, HSPA8, FGA, YWHAQ, HNRNPM, NONO, and EWSR1) (Fig. 4c). The protein abundance rankings based on mass spectrometry results streamlined focus to the heat shock cognate protein 70 (Hsc70) that is encoded by HSPA8. According to the literature, Hsc70 regulates the expression of several PI3K subunits in endothelial cells, which is linked to AKT activation [16]. Therefore, the relationship between Hsc70 expression and PI3K p110 subunit expression was investigated in the TCGA-LIHC database. The findings revealed that Hsc70 mRNA levels correlated with the mRNA levels of PI3K p110α subunit isoforms (Fig. 4d).Immunoblotting was then performed for validation (Fig. 4e).

Surprisingly, GPX8 knockdown or overexpression had no effect on Hsc70 protein levels (Fig. 4f, g). However, suppressing Hsc70 can considerably inhibit the activation of the PI3K-AKT pathway by GPX8 knockdown (Fig. 4f). It has been reported that Hsc70’s subcellular localization is critical for its own function. The Human Protein Atlas (HPA), which is a public database of human proteins, revealed that Hsc70 is enriched in the nucleus in HCC tissues (Fig. 4h). This implies that GPX8 may regulate Hsc70 function by altering its subcellular localization. Following that, immunohistochemical staining of HCC samples in this study validated that Hsc70 protein was overexpressed in the nucleus among HCC cells (Fig. 4h). To verify this hypothesis, nucleoplasmic separation arrays were performed. The results revealed that GPX8 knockdown increased the Hsc70 protein levels in the nucleus while reducing the concentrations in the cytoplasm, indicating that GPX8 knockdown might increase Hsc70 nuclear translocation (Fig. 4i). From these results, it can be extrapolated that GPX8 knockdown increased the nuclear translocation of Hsc70 in HCC cells.

### The combined analysis of GPX8 and Hsc70 provides a potential method for predicting prognosis in HCC

**Fig. 5:**
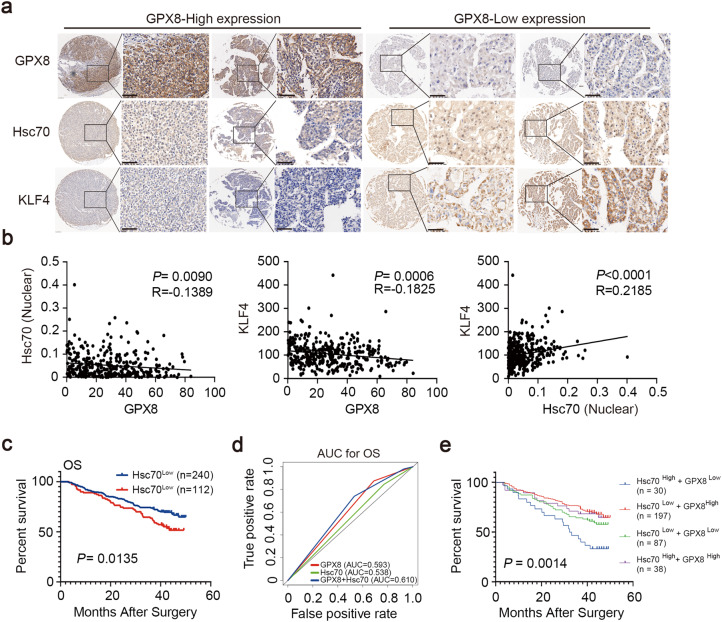
The combination of GPX8 and Hsc70 produced sufficient power for predicting prognosis in HCC. **a** Illustrations of Hsc70, KLF4, and GPX8 IHC staining, 5x Scales, 300 μm, 40 x scales, 50 μm. **b** Correlations between the protein expressions of the Hsc70, KLF4, and GPX8. **c** Kaplan-Meier curve analysis of HCC patients’ OS following curative resection based on Hsc70 expression levels. **d** ROC curve analysis was used to evaluate the prognostic prediction capabilities of Hsc70, GPX8, or combinations for OS. **e** Kaplan-Meier curve analysis of Hsc70-GPX8 combinations after curative resection

HCC microarrays were used to further validate the link between GPX8 and Hsc70 in clinical samples (Fig. 5a). According to the results from immunohistochemical staining, Hsc70 nuclear positivity and cytoplasmic staining were both relatively low and weak in tissues of patients with high GPX8 expression. In the meantime, KLF4 expression was also observed to be low (Fig. 5a left). On the contrary, the Hsc70 nuclear-positive rate and the KLF4 expression were both higher in the tissues with low GPX8 expression (Fig. 5a right). Further statistical analysis revealed a significant negative correlation between the protein expression levels of GPX8 and both the nuclear-positive rate of Hsc70 (*P* = 0.0090) and the expression levels of KLF4 (*P* = 0.0006) (Fig. 5b). Positive correlation was noted between the expression levels of KLF4 and the nuclear-positive rate of Hsc70 (*P* < 0.0001) (Fig. 5b). A high Hsc70 nuclear-positive rate indicated a poor prognosis for overall survival and relapse-free survival in HCC patients (*P*_OS_ = 0.0135, *P*_RFS_ = 0.0410), (Fig. 5c). The Chi-square analyses also revealed that the positive rate of Hsc70 was also significantly positively linked with tumor size (*P* = 0.0049) and BCLC stage (*P* = 0.0052), (Supplementary Table S3).

Subsequently, we have the hypothesis that GPX8 and Hsc70 combination analysis might be a stronger approach for predicting the prognosis of HCC patients. To verify this, the HCC patients were divided into four sub-groups as follows: (i) GPX8-high and HSC70-high (n = 38); (ii) GPX8-high and HSC70-low (n = 197); (iii) GPX8-low and HSC70-high (n = 30); and (iv) GPX8-low and HSC70-low (n = 87). Analyzing the ROC curve revealed that combining GPX8 and Hsc70 improved the identification of patients with a poor prognosis (Fig. 5d, OS: GPX8 0.593, Hsc70 0.538, 0.610). The Kaplan-Meier analysis showed that the GPX8 Low + Hsc70 High group had a poor prognosis for overall survival, while the GPX8 High + Hsc70 Low group had the best prognosis among the four groups (Fig. 5e, *P* = 0.0014). In general, the findings of this study suggest that this combination is a potential method for predicting a patient’s prognosis for HCC, and that the link between GPX8 and Hsc70 has been proved in clinical samples.

## Discussion

**Fig. 6:**
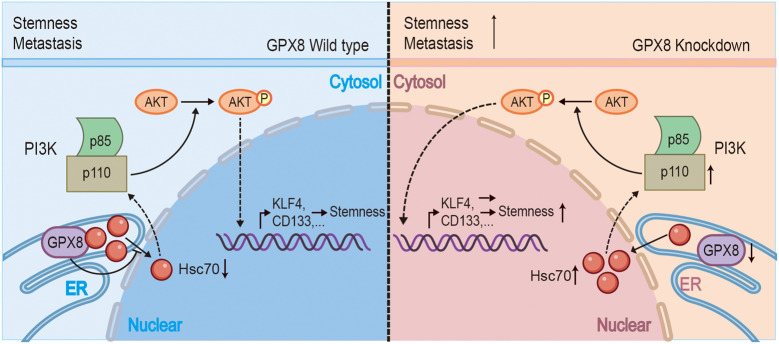
The signaling pathway diagram involved in this study. This figure illustrates how GPX8 affects the translocation of Hsc70 in cell components, leading to altered AKT activation and tumor malignancy

This research was driven by the need to gain better understanding of GPX8, considering its importance in cellular processes and multiple roles in cancer biology[8–11, 17–21]. In HCC, we discovered that GPX8 depletion was associated with a poor clinical prognosis and a more aggressive tumor malignant phenotype, and we created a signaling pathway diagram to help explain our findings (Fig. 6). We hypothesize that GPX8 depletion alters the translocation of Hsc70, triggering the transcription of PI3K p110α within the AKT signaling pathway and enhancing HCC stemness and migration phenotypes.

As a member of the Glutathione Peroxidase family which play a vital role in maintaining redox balance, GPX8 deficiency might induce oxidative stress in HCC cells, and further enhanced the sensitivity to chemotherapeutics. However, in this study, GPX8 downregulation was observed to activate multiple pathways, including the PI3K-AKT, WNT, MAPK, chemokine signaling, and those regulating stem cell pluripotency. Notably, the interaction of AKT, WNT, and MAPK pathways, which have significant implications on cancer stem cell regulation and the maintenance of aggressive cancer phenotypes, is particularly evident in colorectal cancer, glioblastoma, and hepatocellular carcinoma [22–24]. Based on the results from phosphorylated kinase microarrays, we identified that the primary pathway affected by GPX8 is the AKT pathway, which is a well-established signaling cascade that is implicated in cell proliferation, survival, and growth, usually activated in the progression of cancer [25]. These data suggest that GPX8 might play multiple roles in HCC progression and chemotherapy resistance.

The IP experiments identified Hsc70 as the primary molecule that interacts with GPX8, not at the expression level, but by modulating its translocation in cell components. Hsc70 is primarily expressed in the cytoplasm of normal cells and functions as a constitutively expressed protein, implying that its expression level remains consistent across all cells [26]. Hsc70 is known for both its molecule chaperone activity and its function in protecting cells from stress [27, 28]. Previous studies have reported that Hsc70 plays a significant role in endothelial cells (ECs) via the phosphatidylinositol 3-kinase/AKT pathway [16]. We also observed the Hsc70 nuclear enrichment phenomenon in HCC tissues (Fig. 4h). These data suggest the existence of a unique tumor biological process in HCC, where Hsc70 regulates AKT-related pathways by translocating to the nucleus. Thess findings proposed GPX8 and nuclear localization of Hsc70 as novel potential molecular targets for HCC therapeutics.

The present study is not without limitations. While it offers valuable insights, the precise mechanisms by which GPX8 influences Hsc70’s translocation and the broader implications for oxidative stress management within the tumor microenvironment are still yet to be fully elucidated. Additionally, It remains to be fully understood which specific molecular process Hsc70 uses to control the transcriptional level of p110α in the nucleus, though it represents a promising direction for further investigation. Therefore, Finding better therapeutic targets for HCC may be facilitated by a deeper understanding of these hypothesized mechanisms.

In summary, this study contributes to the knowledge regarding GPX8’s role in HCC, thereby presenting it not only as a prognostic biomarker but also as a potential therapeutic target. The interactions between GPX8, Hsc70, and the PI3K-AKT signaling pathway open new avenues for targeted therapies, which could be applied to managing HCC. Future studies should focus on the mechanistic details of GPX8’s regulatory functions as well as the potential therapeutic application of these insights.

## Electronic supplementary material

Below is the link to the electronic supplementary material.

Supplementary Material 1

## Data Availability

All data that support the findings of this study are available from the corresponding authors upon reasonable request.

## Declarations

### Dual publication

The results/data/figures in this manuscript have not been published elsewhere, nor are they under consideration (from you or one of your Contributing Authors) by another publisher.
