# Supplementary material for: Downregulation of GPX8 in hepatocellular carcinoma: impact on tumor stemness and migration
Source: Cell Oncol (Dordr). 2024 Apr 12;47(4):1391–403. doi: 10.1007/s13402-024-00934-w (PMC11322209; doi:10.1007/s13402-024-00934-w)
Supplement: Supplementary file 1 — Supplementary Material 1 [file 13402_2024_934_MOESM1_ESM.docx]

# Supplemental Materials and Methods

## EdU (5-Ethynyl-2'-deoxyuridine) Incorporation Assay

The EdU incorporation assay was conducted to evaluate cell proliferation. EdU is a thymidine analog that gets incorporated into DNA during active DNA synthesis, and its detection allows for the direct measurement of cells in the S-phase of the cell cycle.

Cells were seeded into a 96-well plate at a density of 1x10^4 cells/well and were allowed to adhere for 24 hours. Post-adhesion, the cells were treated as per the experimental conditions.

Following the treatment period, 5-ethynyl-2'-deoxyuridine (EdU) was added to the culture medium at a final concentration of 10 µM and incubated for 2 hours. After incubation, the cells were fixed with 4% formaldehyde for 15 minutes at room temperature and then permeabilized with 0.5% Triton X-100 in PBS for 20 minutes.

The incorporated EdU was detected using a Click-iT EdU assay kit as per the manufacturer's instructions.

## Wound healing assay

Wound healing assay was performed to assess cell migration and wound healing capacity. Firstly, cells were seeded in a 6-well plate at an appropriate density to reach near confluence after 24 hours of growth in a standard culture medium.

After reaching confluence, a sterile 200 µL pipette tip was used to create a straight scratch (i.e., a "wound") in the cell monolayer. The detached cells and debris were removed by washing the wells gently with phosphate-buffered saline (PBS). Fresh medium without serum was then added, and the cells were allowed to migrate into the scratch area.

Photographs of the scratch were taken at time zero (immediately after the scratch was made) and at subsequent time points (typically 24 and 48 hours after scratching), using an inverted microscope. The images were then analyzed to measure the width of the scratch at different time points, and the percentage of wound closure was calculated.

## Supplementary Table

Table S1 Correlation between GPX8 expression and clinical characteristics in 354 HCC patients.

|  | **GPX8 ^Low^**  **(n = 118)** | **GPX8 ^High^**  **(n = 236)** | ***P*-value** |
| --- | --- | --- | --- |
| **Age (years)** |  |  |  |
| Mean (SD) | 52.6 (10.5) | 52.5 (10.3) | 0.931 |
| Median [Min, Max] | 52.0 [30.0, 75.0] | 53.0 [30.0, 75.0] |  |
| **Sex** |  |  |  |
| Female | 19 (16.1%) | 29 (12.3%) | 0.41 |
| Male | 99 (83.9%) | 207 (87.7%) |  |
| **HBsAg** |  |  |  |
| Negative | 25 (21.2%) | 46 (19.5%) | 0.814 |
| Positive | 93 (78.8%) | 190 (80.5%) |  |
| **Liver Cirrhosis** |  |  |  |
| No | 18 (15.3%) | 39 (16.5%) | 0.878 |
| Yes | 100 (84.7%) | 197 (83.5%) |  |
| **ALB** |  |  |  |
| Mean (SD) | 4.34 (0.547) | 4.40 (0.455) | 0.351 |
| Median [Min, Max] | 4.35 [2.80, 6.50] | 4.40 [3.10, 5.70] |  |
| **ALT** |  |  |  |
| Mean (SD) | 53.7 (70.4) | 49.9 (62.1) | 0.617 |
| Median [Min, Max] | 39.5 [10.0, 561] | 37.0 [8.00, 665] |  |
| **GGT** |  |  |  |
| Mean (SD) | 92.6 (108) | 81.0 (75.0) | 0.295 |
| Median [Min, Max] | 61.0 [7.00, 757] | 56.5 [10.0, 648] |  |
| **AFP** |  |  |  |
| ≤20 | 38 (32.2%) | 104 (44.1%) | **0.0422** |
| >20 | 80 (67.8%) | 132 (55.9%) |  |
| **Tumor Number** |  |  |  |
| Single | 106 (89.8%) | 197 (83.5%) | 0.149 |
| Multiple | 12 (10.2%) | 39 (16.5%) |  |
| **Tumor Size** |  |  |  |
| ≤5 cm | 76 (64.4%) | 158 (66.9%) | 0.721 |
| >5 cm | 42 (35.6%) | 78 (33.1%) |  |
| **Differentiation** |  |  |  |
| I/II | 84 (71.2%) | 173 (73.3%) | 0.768 |
| III | 34 (28.8%) | 63 (26.7%) |  |
| **MVI** |  |  |  |
| No | 83 (70.3%) | 168 (71.2%) | 0.967 |
| Yes | 35 (29.7%) | 68 (28.8%) |  |
| **BCLC Stage** |  |  |  |
| BCLC-A | 72 (61.0%) | 138 (58.5%) | 0.731 |
| BCLC-B | 46 (39.0%) | 98 (41.5%) |  |
| **CNLC stage** |  |  |  |
| I | 80 (67.8%) | 154 (65.3%) | 0.721 |
| II+III | 38 (32.2%) | 82 (34.7%) |  |

Table S2 Univariate and multivariate analysis of 354 HCC patients.

|  | **Univariate** | |  | **Multivariate** | |
| --- | --- | --- | --- | --- | --- |
| **Characteristics** | **HR**  **(95% CI for HR)** | ***P-*Value** |  | **HR**  **(95% CI for HR)** | ***P*-Value** |
| **GPX8** | 0.59 (0.42-0.83) | **0.00240** |  | 0.57(0.39-0.75) | **0.00180** |
| **Age** | 1 (0.98-1) | 0.69720 |  |  |  |
| **Sex** | 1.1 (0.64-1.8) | 0.82740 |  |  |  |
| **ALB** | 0.42 (0.29-0.61) | **<0.00001** |  | 0.38(0.2-0.56) | **<0.00001** |
| **ALT** | 1 (1-1) | 0.96200 |  |  |  |
| **HBsAg** | 1.5 (0.91-2.3) | 0.11470 |  |  |  |
| **AFP** | 1.6 (1.1-2.3) | **0.01690** |  | 1.43(1.24-1.62) | 0.05760 |
| **Tumor Number** | 1.5 (0.97-2.3) | 0.07140 |  |  |  |
| **Tumor Size** | 3.3 (2.4-4.7) | **<0.00001** |  | 1.41(1.07-1.75) | 0.31030 |
| **Differentiation** | 1.7 (1.2-2.4) | **0.00360** |  | 1.39(1.2-1.58) | 0.08770 |
| **MVI** | 2.1 (1.5-2.9) | **<0.00001** |  | 1.34(1.15-1.53) | 0.12320 |
| **BCLC Stage**  **B vs A** | 3.3 (2.3-4.7) | **<0.00001** |  | 2.52(2.16-2.88) | **0.01030** |
| **CNLC Stage**  **II+III vs I** | 2.2 (1.6-3.1) | **<0.00001** |  | 2.29(1.87-2.71) | 0.05080 |

Table S3 Correlation between the Hsc70 nuclear-positive rate and clinical characteristics in 352 HCC patients.

|  | **Hsc70 ^Low^ (n = 240)** | **Hsc70 ^High^ (n = 112)** | ***P*-value** |
| --- | --- | --- | --- |
| **Age (years)** |  |  |  |
| Mean (SD) | 53.0 (10.3) | 51.8 (10.4) | 0.328 |
| Median [Min, Max] | 53.0[30.0,75.] | 52.5[30.0,75.0] |  |
| **Sex** |  |  |  |
| Female | 33 (13.8%) | 15 (13.4%) | 1.000 |
| Male | 207 (86.2%) | 97 (86.6%) |  |
| **HBsAg** |  |  |  |
| Negative | 53 (22.1%) | 18 (16.1%) | 0.243 |
| Positive | 187 (77.9%) | 94 (83.9%) |  |
| **Liver Cirrhosis** |  |  |  |
| No | 36 (15.0%) | 21 (18.8%) | 0.463 |
| Yes | 204 (85.0%) | 91 (81.2%) |  |
| **ALB** |  |  |  |
| Mean (SD) | 4.39 (0.501) | 4.36 (0.453) | 0.526 |
| Median [Min, Max] | 4.40 [2.80, 6.50] | 4.40[3.20, 5.40] |  |
| **ALT** |  |  |  |
| Mean (SD) | 53.3 (74.8) | 47.1 (36.4) | 0.3 |
| Median [Min, Max] | 37.0 [8.00, 665] | 41.0 [12.0, 279] |  |
| **GGT** |  |  |  |
| Mean (SD) | 85.6 (97.4) | 82.2 (60.7) | 0.682 |
| Median [Min, Max] | 54.5 [7.00, 757] | 64.0 [16.0, 329] |  |
| **AFP** |  |  |  |
| ≤20 | 95 (39.6%) | 46 (41.1%) | 0.882 |
| >20 | 145 (60.4%) | 66 (58.9%) |  |
| **Tumor Number** |  |  |  |
| Single | 204 (85.0%) | 97 (86.6%) | 0.813 |
| Multiple | 36 (15.0%) | 15 (13.4%) |  |
| **Tumor Size** |  |  |  |
| ≤5 cm | 171 (71.2%) | 62 (55.4%) | **0.005** |
| >5 cm | 69 (28.8%) | 50 (44.6%) |  |
| **MVI** |  |  |  |
| No | 172 (71.7%) | 77 (68.8%) | 0.664 |
| Yes | 68 (28.3%) | 35 (31.2%) |  |
| **BCLC Stage** |  |  |  |
| BCLC-A | 155 (64.6%) | 54 (48.2%) | **0.005** |
| BCLC-B | 85 (35.4%) | 58 (51.8%) |  |
| **CNLC stage** |  |  |  |
| I | 160 (66.7%) | 72 (64.3%) | 0.750 |
| II+III | 80 (33.3%) | 40 (35.7%) |  |

Table S4. qRT-PCR Primer Sequences

| **Primer Name** | **Primer Sequence** |
| --- | --- |
| GAPDH-F | GTCTCCTCTGACTTCAACAGCG |
| GAPDH-R | ACCACCCTGTTGCTGTAGCCAA |
| GPX8-F | TACTTAGGGCTGAAGGAACTGC |
| GPX8-R | GGCTCCGATTCTCCAAACTGA |
| ACTB-F | CATGTACGTTGCTATCCAGGC |
| ACTB-R | CTCCTTAATGTCACGCACGAT |
| KLF4-F | CCCACATGAAGCGACTTCCC |
| KLF4-R | CAGGTCCAGGAGATCGTTGAA |
| EPCAM-F | AATCGTCAATGCCAGTGTACTT |
| EPCAM-R | TCTCATCGCAGTCAGGATCATAA |
| CD133-F | AGTCGGAAACTGGCAGATAGC |
| CD133-R | GGTAGTGTTGTACTGGGCCAAT |

This table provides the primer sequences used for quantitative real-time PCR (qRT-PCR) in the study. The forward (F) and reverse (R) sequences for each primer pair are provided, corresponding to the genes for glyceraldehyde 3-phosphate dehydrogenase (GAPDH), glutathione peroxidase 8 (GPX8), beta-actin (ACTB), Kruppel-like factor 4 (KLF4), epithelial cell adhesion molecule (EPCAM), and CD133.

Table S5 Antibodies in this research

| **Antibodies** | **厂家及货号** |
| --- | --- |
| Hsc70 | Beyotime Rabbit Monoclonal Antibody AF1132 |
| GPX8 | Abcam Rabbit polyclonal Antibody ab183664 |
| KLF4 | CST StemLight™ iPS Cell Reprogramming Antibody Kit #9092 |
| OCT4 |  |
| CD133 | CST Rabbit mAb #64326 |
| Anti-Flag | CST Mouse mAb #8146 |
| p110α | CST Rabbit mAb #4249 |
| ACTB | CST Rabbit mAb #4970 |
| AKT | CST Phospho-Akt Pathway Antibody Sampler Kit #9916 |
| p-AKT |  |
